# Effect of environmental and cultural conditions on medium pH and explant growth performance of Douglas-fir ( *Pseudotsuga menziesii*) shoot cultures

**DOI:** 10.12688/f1000research.5919.2

**Published:** 2015-05-08

**Authors:** Chien-Chih Chen, Rick Bates, John Carlson

**Affiliations:** 1 Department of Genetics and Biochemistry, Clemson University, Biosystems Research Complex, Clemson, SC, 29634, USA; 2 Department of Plant Science, The Pennsylvania State University, University Park, PA, 16802, USA; 3 Department of Ecosystem Science and Management, The Pennsylvania State University, University Park, PA, 16802, USA

**Keywords:** medium pH, Pseudotsuga menziesii, Douglas-fir, micropropagation, 2-(N-morpholino)ethanesulfonic acid, MES

## Abstract

The medium pH level of plant tissue cultures has been shown to be essential to many aspects of explant development and growth. Sensitivity or tolerance of medium pH change *in vitro* varies according to specific requirements of individual species. The objectives of this study are to 1) determine medium pH change over time in storage conditions and with presence of explants, 2) evaluate the effects of medium pH change on explant growth performance and 3) assess the effects of adding a pH stabilizer, 2-(N-morpholino)ethanesulfonic acid (MES) that is commonly used in Douglas-fir micropropagation medium. Vegetative buds were collected in the spring before breaking dormancy from juvenile and mature donor trees for conducting these evaluations. Medium, with or without MES, was pre-adjusted to five pH levels before adding MES, agar and autoclaving. Medium pH changes and explant growth parameters were measured at eight different incubation times. Overall, MES provided a more stable medium pH, relative to starting pH values, under both light and dark storage conditions as well as with presence of explants. A general trend of decreasing medium pH over time was found comparing explants from juvenile and mature donor genotypes. Explant height and weight growth increased over time, but differ among explants from juvenile and mature donor genotypes. Our findings suggest that a 21-day subculture practice may best sustain medium freshness, medium pH level and desirable explant growth.

## Introduction

The Christmas tree industry plays an important role within Pennsylvania agriculture as well as across the nation. The goal of this micropropagation project was to develop a true-to-type clonal propagation system to alleviate the cost of tree-to-tree variation from conventional seedling propagation. Understanding plant materials and their growing conditions may provide better assistance for later developmental stages in tissue culture.

The medium pH of plant tissue cultures has been shown to be very important to many aspects of explant development and growth. Sensitivity or tolerance to medium pH change *in vitro* varies according to specific requirements of individual species. Similar to soil pH, medium pH level may influence nutrient uptake ( Ramage & Williams, 2002), cellular pH adjustment ( Ballarin-Denti & Antoniotti, 1991), rooting and cellular growth ( de Klerk *et al.*, 2008; Leifert *et al.*, 1992), plant gene expression and transcriptional pH responses in roots ( Lager *et al.*, 2010), and the efficiency of *Agrobacterium*-mediated transformation ( Ogaki *et al.*, 2008; Rai *et al.*, 2012). Medium pH also can act to facilitate or inhibit nutrient availability in the medium, such as ammonium uptake *in vitro* being facilitated with a stable pH of 5.5 ( Thorpe *et al.*, 2008).

Medium pH fluctuations may be attributed to medium components, autoclaving, ion exchange, and environmental conditions. Medium components may modify pH prior to and after autoclaving ( Owen *et al.*, 1991; Skirvin *et al.*, 1986). Organic, inorganic salts, amino acids, vitamins, sucrose, gelling agents, and plant growth regulators are the common components added to tissue culture medium. Williams *et al.* (1990) reported that adding agar significantly elevated medium pH prior to autoclaving when pre-adjusted pH ranged from 3.5 to 5.5 in MS medium ( Murashige & Skoog, 1962), while less pH increment was found for pre-adjusted medium pH ranging from 5.5 to 7.0, and pH decreased for pre-adjusted medium pH ranging from 7.0 to 8.0. In contrast, post-autoclaving medium pH increased for pre-adjusted pH of 3.5–4.5, but a more significant medium pH decrease was observed with pre-adjusted pH of 5–8. Additions of synthetic or natural organic acids generally increase medium buffering ability ( Thorpe *et al.*, 2008). Organic compounds such as 2-(N-morpholino)ethanesulfonic acid (MES) are known to help maintain suitable medium pH range for explant development ( de Klerk *et al.*, 2008; Parfitt *et al.*, 1988; Yuan *et al.*, 2012). MES and vitamin additions were also found to enhance embryo growth during the initiation stage of Douglas-fir ( *Pseudotsuga menziesii* (Mirb.) Franco) (Franco 1950) somatic embryogenesis ( Pullman *et al.*, 2005).

After placing explants on the medium, medium pH fluctuations have also been reported for various species. Medium pH of loblolly pine ( *Pinus taeda*) grown in liquid suspension medium showed subsequent pH decrease from 5.5 to 4.6 within 5 days of incubation followed by pH increase to 6.0 in 19 days of incubation ( Pullman *et al.*, 2005). Significant medium pH decrease was also found after placing mulla mullas ( *Ptilotus exaltatus*) shoots on MS medium for 4 weeks ( Williams *et al.*, 1990) and an U shaped curve of medium pH fluctuation was found in shoot tip culture of crabapple ( *Malus* sp. cv. Almey) and pear ( *Pyrus communis* L. cv. Seckel) on MS medium ( Singha *et al.*, 1987). According to Skirvin *et al.* (1986) and Thorpe *et al.* (2008), explant nutrient absorption *in vitro* is a function of ion exchange where deposition of free hydrogen ions (H ^+^) and hydroxyl ion (OH ^-^) in the medium may contribute to acidic or alkaline medium pH. In contrast, photooxidation induced chelating events that bind free iron, reducing iron availability, may also influence medium pH ( Hangarter & Stasinopoulos, 1991). Secretion of plant secondary metabolites into culture medium is common *in vitro* ( Dörnenburg & Knorr, 1995) but its role and function in altering medium pH and nutrient absorption are not clear.

Medium pH fluctuations can involve many factors, and can eventually become problematic for tissue culture. Douglas-fir shoot culture can be grown in a modified version (mDCR) of Douglas-fir cotyledon revised medium (DCR, Gupta & Durzan, 1985; Gupta & Durzan, 1987a). However, the interactions between the species and its growing medium are not well understood. To ensure an optimal shoot culture development and provide high quality shoots for later development of rooting protocol, medium pH can be a key indicator in determining optimal subculture time. Medium pH may be further utilized as a diagnostic tool for some abnormal growth symptoms, such as necrosis, caused by low pH induced nutrient deficiency. The study of medium pH effects may also be extended to explant development and nutrient relationships *in vitro* (Singha *et al.*, 1990). Hence, the objectives of this study are to 1) determine medium pH change over various times under storage conditions and in the presence of explants, 2) evaluate the effects of prolonged culturing on medium pH and explant growth performance, and 3) assess the effects of addition of a pH stabilizer, MES to Douglas-fir micropropagation medium.

## Materials and methods

Vegetative buds from juvenile (HF205 and HF210) and mature (PS-2) donor trees collected in the spring of 2006 were utilized for this study. These bud samples were collected prior to breaking dormancy. To classify juvenility, Douglas-fir trees of Lincoln seed source planted 10 years previously and not yet producing cones at the Penn State Horticulture Research Farm of Russell E. Larson Agricultural Research and Education Center at Rock Springs were the selected as juvenile donors. The selected mature donor tree, an elite Christmas tree genotype resulting from a previous genetics study conducted by Gerhold (1984), was over 40 years old and growing in a seed orchard located on the Penn State University golf course. These bud samples were stored in a 4°C cold room until culture initiation. Preparation, sterilization, and dissection of collected bud samples followed Traore *et al.* (2005).

This study consisted of a four-factor factorial design with three replications for each factor combination. Two juvenile genotypes, HF205 and HF210, and one mature genotype, PS-2 were entered for evaluations. The basal medium was mDCR, a modified version of the MS basal salts developed for Douglas-fir by Gupta & Durzan (1985 and 1987a) which we further modified and classified as mDCR. A comparison of the components between DCR vs. mDCR is provided in Table 1. Two types of media were used including mDCR only and mDCR with 2 g/L of MES (mDCR+MES) (M3671, Sigma-Aldrich, St. Louis, MO, USA). Levels of media pH were pre-adjusted to 3.6, 5.1, 5.7, 6.3, and 7.8 before adding 7 g of agar, MES, and autoclaving. This wide range of pre-adjusted medium pH levels would allow assessments of medium buffering ability and explant growth response and adjustment after placing onto corresponding medium types. These pre-adjusted medium pH levels were also confined within the pH levels that will not interfere with gel solidification. Due to limited plant materials, the addition of one previously reported concentration of MES ( de Klerk *et al.*, 2008; Höfer *et al.*, 1999; Parfitt *et al.*, 1988; Wilson *et al.*, 1989) served as the basis for comparison in aforementioned assessment needs. Five surface-sterilized dissected vegetative buds from each genotype were placed on each treatment combination. Controls consisted of medium without the presence of explants, at each pH level. They were placed in full dark versus light in 25°C growth chambers. For treatments, explants were dissected and placed into mDCR versus mDCR+MES media for incubation for 1, 3, 5, 7, 14, 21, 28, and 35 days.

**Table 1.** Medium formulation comparison of DCR vs. mDCR.

Macronutrient			
Molecular formula	Compound name	Amount to add (mg/L)	
		DCR	mDCR
KNO _3_	Potassium nitrate	340.000	1000.000
(NH _4_) _2_SO _4_	Ammonium sulfate	-	200.000
KCl	Potassium chloride	-	300.000
MgSO _4_ · 7H _2_O	Magnesium sulfate heptahydrate	370.000	250.000
CaCl _2_ · 2H _2_O	Calcium chloride dihydrate	85.000	150.000
NaH _2_PO _4_ · H _2_O	Sodium phosphate monobasic monohydrate	-	90.000
Na _2_HPO _4_ · 7H _2_O	Sodium phosphate dibasic heptahydrate	-	30.000
Ca(NO _3_) _2_ · 4H _2_O	Calcium nitrate tetrahydrate	556.000	-
KH _2_PO _4_	Potassium dihydrogen phosphate	170.000	-
NH _4_NO _3_	Ammonium nitrate	400.000	-
Micronutrient			
ZnSO _4_ · 7H _2_O	Zinc sulfate heptahydrate	8.600	3.000
H _3_BO _3_	Boric acid	6.200	3.000
CuSO _4_ · 5H _2_O	Cupric sulfate pentahydrate	0.250	0.125
FeSO _4_ · 7H _2_O	Ferrous sulfate heptahydrate	27.800	13.900
MnSO _4_ · H _2_O	Manganous sulfate monohydrate	22.300	5.300
C _10_H _14_N _2_Na _2_O _8_ · 2H _2_O	EDTA disodium salt dihydrate	37.300	18.700
KI	Potassium iodide	0.830	0.375
CoCl _2_ · 6H _2_O	Cobalt chloride hexahydrate	0.025	0.125
Na _2_MoO _4_ · 2H _2_O	Sodium molybdate dihydrate	-	0.125
NaMoO _4_ · 2H _2_O	Sodium molybdenum oxide dihydrate	0.250	-
NiCl _2_	Nickel(II) chloride	0.025	-
Vitamin			
C _6_H _12_O _6_	*myo*-Inositol	200.000	1.000
C _6_H _5_NO _2_	Nicotinic Acid	0.500	0.100
C _12_H _17_ClN _4_OS · HCl	Thiamine HCl	1.000	1.000
C _8_H _11_NO _3_ · HCl	Pyridoxine HCl	0.500	0.100
NH _2_CH _2_COOH	Glycine	2.000	-

Note: Douglas-fir cotyledon revised media (DCR, Gupta & Durzan, 1985, Gupta & Durzan, 1987a); modified Douglas-fir cotyledon revised media (mDCR)

During the dissection process, measurements of samples were taken for initial bud weight (mg) and petri dish (PD) weight (mg) with solidified media. After each treatment and incubation time, final media pH, final PD weight (mg), and explant final weight (mg) were recorded. Explant weight change (mg) was obtained by subtracting the initial bud weights from final explant weights. Medium pH was measured at five positions in the plates, between the explants, using a Thermo Orion PerpHecT pH meter (Thermo Fisher Scientific Inc., Waltham, MA, USA). Data analyses were performed using Minitab (Minitab Inc., State College, PA, USA), and graphs were generated by SigmaPlot (Systat Software Inc., Chicago, IL, USA), including ANOVA General Linear Model (GLM), Tukey Honestly Significant Difference test (HSD), and linear regression with significance set at p<0.05.

## Results

Media pH and explant weight changes over incubation times for Douglas fir (Pseudotsuga menziesii) shoot cultures grown on different media Dataset 1: Data column A through E are Conditions (incubation conditions as in Light or dark at 25C), Medium Type (mDCR refers to a modified version of Douglas-fir cotyledon revised medium, and mDCR+MES refers to mDCR medium with addition of 2-(N-morpholino)ethanesulfonic acid), Initial pH (pre-adjusted levels of medium pH), Incubation Time (days), and pH Measurement (post-treatment medium pH measurement).
Dataset 2: Dataset 2 is the comparison of Pre- and Post-autoclaved medium pH measurement (data column A and B, respectively).
Dataset 3: Dataset 3 column A through E are Genotype, Initial pH (pre-adjusted medium pH), Medium Type (mDCR refers to a modified version of Douglas-fir cotyledon revised medium, and mDCR+MES refers to mDCR medium with addition of 2-(N-morpholino)ethanesulfonic acid), pH Measurement (medium pH changes according to treatments), and Incubation Time (days).
Dataset 4: Dataset 4 consists of individual bud weight changes prior to and post treatments according to each genotype. Column A through G are Genotype, Initial pH (pre-adjusted medium pH), Medium Type (mDCR refers to a modified version of Douglas-fir cotyledon revised medium, and mDCR+MES refers to mDCR medium with addition of 2-(N-morpholino)ethanesulfonic acid), Initial Bud Weight (mg), Final Bud Weight (mg), Bud Weight Increment (mg), and Incubation Time (days). Click here for additional data file. Copyright: © 2015 Chen CC et al. 2015 Data associated with the article are available under the terms of the Creative Commons Zero "No rights reserved" data waiver (CC0 1.0 Public domain dedication).

### Culture medium pH change after autoclaving and storage

**Figure 1.**
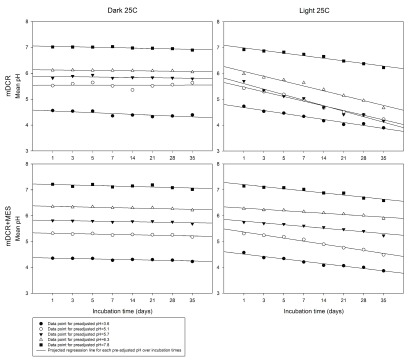
Media pH changes over incubation times from mDCR and mDCR+MES media incubated under dark and light conditions. Medium pH was preadjusted to 3.6, 5.1, 5.7, 6.3, and 7.8 prior to adding 7 g/L of agar, MES and autoclave. Medium was incubated in a growth chamber with full light or darkness at 25°C until it reached incubation requirement. Post-autoclaving pH was recorded using a pH meter at each incubation time. Data points represent mean pH (n=5 for each data point), and were fitted with linear regression lines. Please see Dataset 1 for the raw data.

After autoclaving, media pH shifts were found according to each pre-adjusted media pH. From 100 samples, mDCR medium showed a greater extent of pH fluctuations than mDCR+MES post-autoclaving. Media initially set at pH 3.6, 5.1, and 5.7 showed increased pH for both media types (pH changes of 0.83, 0.58, 0.17 and 0.76, 0.22, 0.11 for mDCR and mDCR+MES, respectively) whereas medium initially at pH 7.8 showed decreased pH (-0.66 and -0.59 for mDCR and mDCR+MES, respectively). Medium of pre-adjusted pH 6.3 showed decreased pH in mDCR (-0.11) but increased pH for mDCR+MES (0.05). In general, dark storage was better to maintain stable media pH than storage in light. Overall, media pH was 5.8 (n=400) when kept in the dark condition compared to pH 5.4 (n=400) in the light ( *P*=0.000). Over the incubation times tested, media pH showed only a slight decreasing trend in the dark storage condition. In contrast, media pH had a stronger decreasing trend when incubated in the light. The mDCR+MES medium maintained media with less pH change over the incubation times when compared to mDCR media in the light ( Figure 1).

### Medium pH change with explants

**Figure 2.**
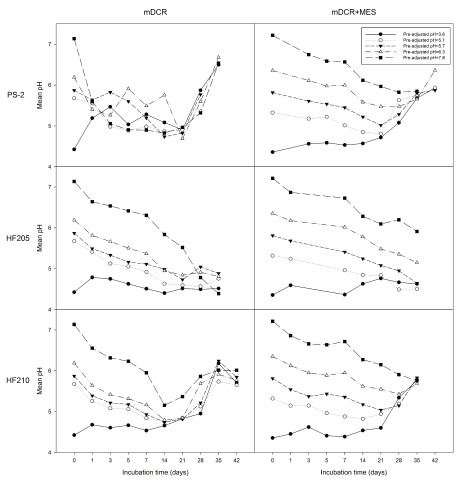
Medium pH changes over incubation times from three genotypes in mDCR and mDCR+MES media. Genotypes included one mature genotype, PS-2 and two juvenile genotypes, HF205 and HF210. After surface sterilization and dissection, inner vegetative buds from above three genotypes were placed on either mDCR or mDCR+MES medium for incubation up to 42 days. These buds were incubated in a growth chamber at 25°C with light regime adjusted to 16-hour light followed by 8-hour darkness each day. Medium pH was recorded using a pH meter when sample reached each incubation requirement where five pH values were recorded within each petri dish. Data points represent mean pH. Please see Dataset 2 and Dataset 3 for the raw data.

After placing explants into the media, the pH of the medium was significantly influenced by all factors - genotype, media type, initial pH level, and incubation time (all *P*=0.000). Overall medium pH was 5.45 from medium incubated with genotype PS-2 explants (n=1,355), which was significantly greater than 5.41 and 5.19 pH for media incubated with explants from genotypes HF210 (n=1,384) and HF205 (n=950), respectively. The medium pH from mDCR+MES (n=1,805) was significantly greater than the pH of mDCR only medium (n=1,884) (5.45 vs. 5.28, respectively) ( *P*=0.000). Decreasing media pH over incubation time and variation among genotypes were both observed. Media with addition of MES was better able to maintain stable media pH up to 21 days of incubation from each of the 5 different initial pH levels ( Figure 2). Regardless of initial pH level, incubation time had a strong significant effect on media pH ( *P*=0.000). Overall for both types of media, mean medium pH showed the lowest value of 5.04 (n=435) at 21-day of incubation. In contrast, the highest mean medium pH 5.88 was recorded at the day 42 of incubation (n=125). Overall medium pH at each initial pH (3.6, 5.1, 5.7, 6.3, and 7.8) showed significant differences between each other including 4.90 (n=744), 5.08 (n=740), 5.30 (n=745), 5.57 (n=740), and 5.99 (n=720), respectively. The media pH stabilizer MES demonstrated its ability to prevent media pH from dropping at the higher or lower ends of initial pH levels. Within individual genotypes, both media type, and incubation time showed significant effects on media pH for all genotypes ( *P*=0.000).

### The effect of MES on explant growth

**Figure 3.**
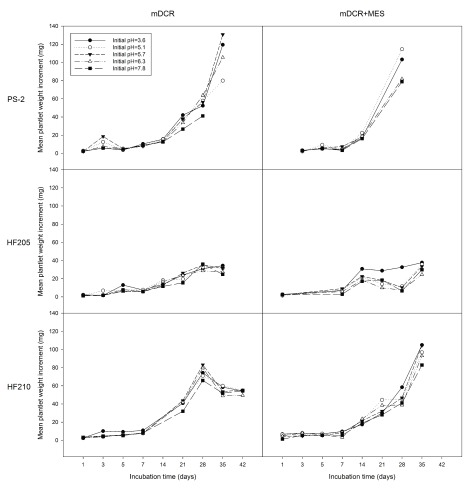
Mean explant weight increment changes (mg) among genotypes and medium types at each initial pH level. Genotypes included one mature genotype, PS-2, and two juvenile genotypes, HF205 and HF210. After surface sterilization and dissection, inner vegetative buds from above three genotypes were placed on either mDCR or mDCR+MES medium at each initial pH level (3.6, 5.1, 5.7, 6.3, and 7.8) for incubation up to 42 days. The buds were incubated in a growth chamber at 25°C with light regime adjusted to 16-hour light followed by 8-hour darkness each day. Bud weight change was recorded using an electronic scale when samples reached each incubation time point. Data points represent mean weight increment (mg). Please see Dataset 4 for the raw data.

For explants growth response, genotype ( *P*=0.000), incubation time ( *P*=0.000), and initial pH ( *P*=0.012) showed significant effects on explant weight increment (mg). The addition of MES into the media did not show a significant effect on explant weight increment ( *P*=0.281) ( Figure 3). Overall, genotype HF210 (30.86 mg, n=264) and PS-2 (22.50 mg, n=190) had a significantly greater weight increment than genotype HF205 (13.83 mg, n=205) ( *P*=0.000). Explant weight increment of HF210 did not show a significant difference when compared with PS-2 ( *P*=0.2903). However, a distinct trend in explant weight decrease was observed after 28 days of incubation in mDCR only medium for HF210. Initial medium pH of 3.6 produced a significantly greater bud weight change ( *P*=0.005) than medium pH 7.8 (25.89 vs. 19.23 mg, n=134 vs. 130, respectively). Although an increasing trend of bud explant weight change was observed, bud weight did not show significant increases during the first week of incubation.

**Figure 4.**
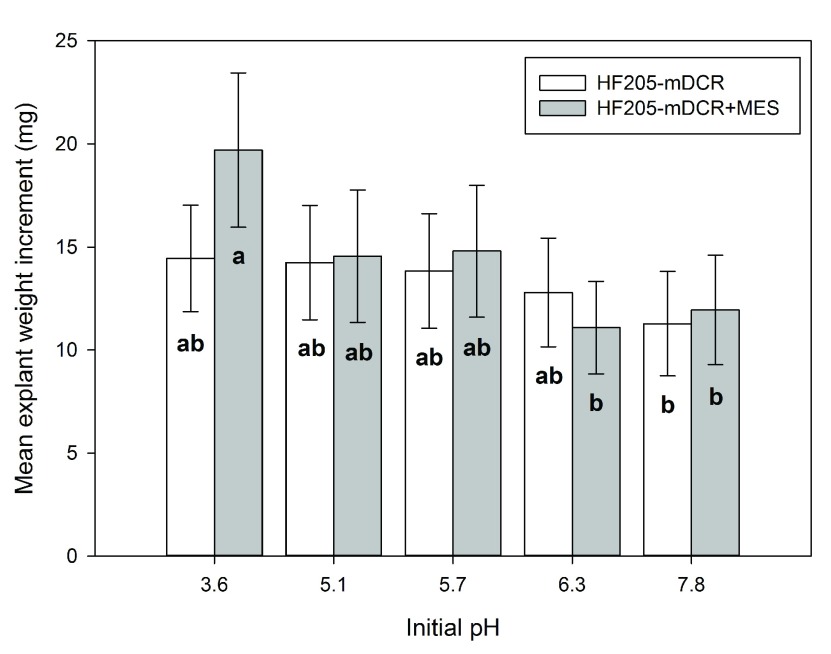
Mean explant weight increment changes (mg) among medium types at each initial pH level from HF205. After surface sterilization and dissection, inner vegetative buds from HF205 were placed on either mDCR or mDCR+MES medium at each initial pH level (3.6, 5.1, 5.7, 6.3, and 7.8) for incubation up to 42 days. These buds were incubated in a growth chamber at 25°C with light regime adjusted to 16-hour light followed by 8-hour darkness each day. Bud weight change was recorded using an electronic scale when sample reached each incubation time point. Vertical bars represent mean weight change (mg)±S.E. Means sharing the same letter indicate non-significant difference between means ( *P*>0.05). Tukey’s (HSD) multiple comparison was used.

**Figure 5.**
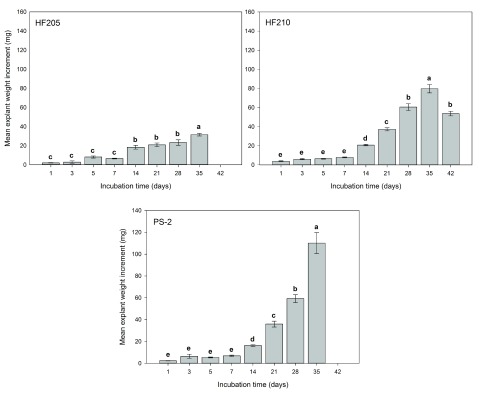
Mean explant weight changes (mg) over incubation times from PS-2, HF205 and HF210. After surface sterilization and dissection, inner vegetative buds from three genotypes were placed on either mDCR or mDCR+MES medium at each initial pH level (3.6, 5.1, 5.7, 6.3, and 7.8) for incubation up to 42 days. These buds were incubated in a growth chamber at 25°C with light regime adjusted to 16-hour light followed by 8-hour darkness each day. Bud weight change was recorded using an electronic scale when sample reached each incubation time point. Vertical bars represent mean weight change (mg)±S.E. Sample sizes according to the incubation time (1–42 days) were for HF205, n=45, 15, 15, 30, 30, 20, 20, and 30, for HF210, n=30, 30, 30, 30, 30, 31, 30, 38, and 15, and for PS-2, n=15, 30, 30, 30, 30, 16, 33, and 6. Means sharing the same letter indicate non-significant difference between means ( *P*>0.05). Tukey’s (HSD) multiple comparison was used. Please see Dataset 4 for the raw data.

Comparing individual genotypes, medium type exhibited non-significant effects on explant weight increment of all three genotypes ( *P*>0.05). For HF205, the weight differences were observed only at the higher and lower ends of the given initial pH levels ( Figure 4). Incubation time showed significant effect on explant weight increment for all three genotypes ( *P*=0.000). For all three genotypes, explant weight did not show any significant differences for the first 7 days of incubation. However afterwards, explant weight growth dramatically increased for genotypes PS-2 and HF210. HF205 showed much less weight increment than the other two genotypes ( Figure 5).

### Morphological observations of explants

After growing in medium for 28-days or more without being subcultured, explants showed various growth deformities such as chlorosis, delayed needle expansion, tip browning, browning of the bottom of explants and surrounding medium, vitrification, and even death. These symptoms occurred especially at the lower and higher ends of the initial pH levels, after prolonged culturing. Regardless of the given initial pH levels, explant growth did not show obvious delay at the early culture stages. The mentioned deformities were only found at the longer times of culturing. Moisture condensation was a common problem in the plate-based tissue culture system. Some of the deformities observed may have been associated with excessive amount of water droplets falling onto the medium surface or coming into contact with the explants.

## Discussion

In general, mDCR medium with MES provided more stability of the pre-adjusted pH values after autoclaving in both the absence and presence of explants in the medium. We observed that after autoclaving, medium pH changed, but mDCR medium with MES showed less medium pH fluctuation than without MES. Storage of medium in the dark resulted in less medium pH fluctuation than storage under light. For mDCR medium incubated with explants, pH showed a gradual decrease that was followed by a sharp increase over the incubation time. However, mDCR+MES medium exhibited a slower decrease in pH or was followed by a convergent medium pH change for all pre-adjusted pH levels. Explant weight gain over time showed an inverse relationship with medium pH change, but also differed between juvenile and mature genotypes. The addition of MES did not show significant influence on explant weight growth. However, a distinct decrease in explant weight growth was observed after 28 days of incubation in the mDCR only medium.

Medium storage is a common practice in tissue culture. The use of premade medium serves two main purposes. One is to hold the medium for a period of time to observe whether any contamination occurs in the medium. This ensures maximum explant growth performance achieved when antibiotics are not present in the medium. The other purpose is to facilitate timely arrangements of routine culture initiations and transfers. Owen *et al.* (1991) demonstrated that light affects post-autoclave medium storage, resulting in reduced medium pH over storage time. Our data confirmed their findings. Medium pH was more stable in the dark storage condition regardless of the presence of MES. The addition of MES could stabilize medium pH under the light condition, in both the presence and absence of explants.

Fluctuation of medium pH can be influenced by many factors. Hydrolysis, enzymatic break down, photooxidation and photolysis on light-sensitive medium components may all contribute to the fluctuation of medium pH. Sucrose hydrolysis often requires splitting of water molecules and breaking glycosidic bonds of the disaccharides. Once the breakage of glycosidic bonds has occurred, hydrogen ions from the splitting of water molecules bind with glucose, whereas hydroxyl groups bind with fructose. Since tissue culture medium often is adjusted to slight acidic conditions (pH 5.2–5.8), autoclaving provides a suitable temperature for catalyzing sucrose hydrolysis. Acid-facilitated autocatalyzed sucrose hydrolysis was reported as being both pH and temperature dependent, where lower pH at a given temperature promotes more sucrose hydrolysis ( Heidt *et al.*, 1952; Wann *et al.*, 1997). The availability of hydrogen ions in the acidic medium solution also depends on the buffering ability of the nutrient components ( Thorpe *et al.*, 2008). Furthermore, carbon sources, the amount of carbohydrates, and gelling agents act together to determine the amount of sucrose hydrolysis and the medium pH after autoclaving. As a result, medium with lower original pH may become higher while medium with higher original pH may become lower to reach equilibrium of the solution.

After explants are introduced into the medium vessels, the sucrose is further converted into monosaccharides inter- and intra-cellularly by invertase or other plant enzymes ( Thorpe *et al.*, 2008). Egger & Hampp (1993) found the optimum activity of soluble acid invertase was at pH 4.1 in developing spruce ( *Picea abies* (L.) Karst.) needles. They also reported that other sucrose synthesis enzymes, sucrose phosphate synthase, and sucrose synthase, were pH dependent for their optimal activities (pH 7.7 and 6.7, respectively). Hence, as explants host numerous biological activities they must balance pH levels accordingly for each of the biochemical reactions. Ion uptake and release become the mechanism by which cells adjust for pH requirements. Dodds & Roberts (1995) attributed the fluctuation of medium pH after autoclaving may be a result of imbalance of anion and cation uptake.

Photochemistry may further induce degradation of photo-sensitive compounds in the medium, and trigger pH fluctuation. Photolysis is an event introduced by photons. For example, when a water molecule receives energy from photons during the photosynthesis process, photolysis occurs to generate electrons, hydrogen ions, and oxygen. If the free hydrogen ions are not bound by other substrates and excreted into the medium, they may cause medium pH fluctuation. Hangarter & Stasinopoulos (1991) reported a light induced Fe-catalyzed photooxidation of EDTA, which caused reduced root growth of the *Arabidopsis thaliana* ecotype Columbia. EDTA, an ion chelator, is considered to be a buffering agent in the tissue culture medium. After photooxidation occurred, formaldehyde and glyoxylic acid are produced, which can be toxic to explants, concomitant with increased chelated ferric oxide that explants can not readily use. This light induced change of buffering ability could definitely alter nutrient availability in the medium, and further affect the fluctuation of medium pH.

MES can be utilized to stabilize medium pH for Douglas-fir micropropagation. MES has been employed in various tissue culture systems to maintain stable medium pH over extended culture times. Park & Son (1992) reported the addition of MES alone in the medium, or together with Dithiothreitol, increased protoplast yield and viability from hybrid poplar protoplast culture system. Similarly, MES and arabinogalactan-protein, alone or combined, were found to maintain suitable medium pH and to enhance embryogenesis in white cabbage ( *Brassica oleracea* var. *capitata*) microspore culture system ( Yuan *et al.*, 2012). For conifer species, MES was also utilized as pH stabilizer for silver fir ( *Abies alba* L.) ( Hartmann *et al.*, 1992), white spruce ( *Picea glauca* (Moench) Voss) ( Wilson *et al.*, 1989) and European larch ( *Larix decidua* Mill.) ( Korlach & Zoglauer, 1995) protoplast culture systems. Protoplasts, being a single cell without cell wall, are probably extremely sensitive to rapid pH changes. The effects of pH changes could be as important for shoot culture or other tissue culture prospects for explant productivity. MES may be especially important to maintain stable medium pH for bulk medium preparation in large scale propagation projects. Further evaluation of MES dose-response relationships and determination of the optimal MES concentration could benefit the development of Douglas-fir micropropagation system, although a wide range of successful MES concentrations have been reported previously for Douglas-fir and other conifers ( Thorpe *et al.*, 2008).

Overall our results suggest that a 21-day subculture practice may be most suitable for maintaining medium freshness, medium pH level, and desirable explant growth for Douglas-fir shoot culture. Explant weight increment across various levels of pre-adjusted medium pH was genotype dependent. A broader evaluation including more genotypes could provide stronger or more specific support for such genotype-culture interactions, although this is a generally well known and expected factor in plant tissue culture ( Thorpe *et al.*, 2008). Typically, explant weight increment exhibited a curvilinear relationship with pH over time *in vitro*. After 28 days we did however observe a decreased weight gain in genotype HF210 on mDCR and a cessation of weight gain in genotype HF205 on both media. Whether this growth decline is associated with nutrient depletion, PGR degradation, medium pH, or a combination of these factors, may be resolved through further evaluations of the Douglas-fir tissue culture system. However, based on medium pH fluctuation after in the presence of explants and morphological observations on the explants, prolonged culture without subculturing could result in unwanted growth complications. Therefore, we would suggest subculturing at 21-days for Douglas-fir as a best management practice. Although there might be specific pH requirements for individual species, explants of Douglas-fir genotypes showed various responses or adaptations to medium pH changes. Some genotypes may be able to tolerate or adapt better to fluctuations in medium pH, and to show continuous growth in a wide range of pH levels. The effects of MES and nutrient acquisition by explants in culture may require further investigations on specific aspects of nutrient dynamics regarding the effects of both medium and explants *in vitro*.

## Data availability

The data referenced by this article are under copyright with the following copyright statement: Copyright: © 2015 Chen CC et al.

Data associated with the article are available under the terms of the Creative Commons Zero "No rights reserved" data waiver (CC0 1.0 Public domain dedication). 

Figshare: http://dx.doi.org/10.6084/m9.figshare.1257689 ( Chen *et al.*, 2014).
