# Supplementary material for: Effect of environmental and cultural conditions on medium pH and explant growth performance of Douglas-fir ( Pseudotsuga menziesii) shoot cultures
Source: F1000Res. 2015 May 8;3:298. Originally published 2014 Dec 8. [Version 2] doi: 10.12688/f1000research.5919.2 (PMC4617322; doi:10.12688/f1000research.5919.2)
Supplement: Peer review history

# Peer review: Referee response for version 2

**DOI:** 10.5256/f1000research.6947.r10838

**Published:** 2015-10-16

**Authors:** Vibha Srivastava

**Affiliations:** 1 Department of Crop, Soil, and Environmental Sciences, University of Arkansas, Fayetteville, USA

I approve this manuscript, and have no further comments.

I have read this submission. I believe that I have an appropriate level of expertise to confirm that it is of an acceptable scientific standard.

# Peer review: Referee response for version 2

**DOI:** 10.5256/f1000research.6947.r10681

**Published:** 2015-10-06

**Authors:** Isabel Arrillaga

**Affiliations:** 1 Departamento de Biología Vegetal, Facultad de Farmacia, Universidad de Valencia, Valencia, Spain

I have read this submission. I believe that I have an appropriate level of expertise to confirm that it is of an acceptable scientific standard.

# Peer review: Referee response for version 1

**DOI:** 10.5256/f1000research.6323.r7619

**Published:** 2015-02-10

**Authors:** Isabel Arrillaga

**Affiliations:** 1 Departamento de Biología Vegetal, Facultad de Farmacia, Universidad de Valencia, Valencia, Spain

The effect of the pH in the culture medium is a very important factor that often is not taken into account. This papers reports on the changes in the medium pH at several times during the tissue culture process and their effect on the explants growth. From this point of view the article is very interesting and deserves to be accepted for publication. Changes in the pH of the culture medium after and before autoclaving and during culture time have been studied in depth (see any of the editions of the book series Plant Propagation by Tissue Culture, George and Sherrington, Exegetics, London).

The paper is well written and data well analyzed. I have found very interesting results as the effect of light on the medium pH during storage that can be of interest for plant tissue colleagues.

Also, I have some questions to address to the authors.

1. Why to use pH below 5?. Most of the culture media are adjusted to a pH from 5,7- to 6,0 before autoclaving.
2. Did authors find any problems with low pH and agar gelification?. In our experience low pH affect media solidification.
3. The effect of MES on medium pH stability is expected.
4. From your data on Figures 3 and 5, I should not agree with your statement “21-day subculture practice may facilitate to sustain medium freshness, medium pH and desirable explant growth” (Abstract).  It is true that pH of the medium increased after 21 of explant culture initiation, but it is also true that irrespective of the medium pH, explants sharply increased weight after 21 days in culture. These results should be emphasized and conclusions rewritten.

I have read this submission. I believe that I have an appropriate level of expertise to confirm that it is of an acceptable scientific standard, however I have significant reservations, as outlined above.

# Response

**Published:** 2015-02-10

**Authors:** Chien-Chih Chen

**Affiliations:** Clemson University, USA

Dr. Arrillaga, thank you so much for your comments. We are now working on the revision of this manuscript addressing reviewers' concerns.

# Response

**Published:** 2015-04-29

**Authors:** Chien-Chih Chen

**Affiliations:** Clemson University, USA

We appreciate your comments. Please find our explanations regarding your concerns with the original version (and how they have been addressed in the new version of the article) below.

1. The reason that we chose the larger range of pH levels was to expand upon observations from our routine practice in tissue culture medium preparation. Especially, this was to understand how Douglas-fir explants would respond to various levels of medium pH *in vitro*, while the 5.7 to 6.0 range derives more from *in vivo* conditions. The medium pH and explant weight increment curves provide detailed records relevant to both storage and tissue culture conditions, which we feel also expands upon the range of observations normally reported.
2. You are correct. Low medium pH can affect agar gelling, and this is associated with types of gelling agents used and other additives (i.e. high concentrations of PEG) as well. We did have an issue with gelling, but not until the pre-adjusted medium pH was below pH 3.6 in our case.
3. We agree that MES is a commonly used buffer in tissue culture.
  Because the pKa for MES is 6.15 at 25 °C, we felt it was necessary to observe what the buffering capacity of MES was with starting pH levels far from 6.15 that we wanted to investigate in medium under storage and with explants. If MES is added, what medium pH can be maintained, and for what duration, at such extreme pH levels under our test conditions?
4. This is a very good point. As you can see from Fig. 3 and 5, explant weight increment according to various levels of pre-adjusted medium pH was genotype dependent. A broad evaluation including more genotypes would provide a more strong indication. Usually, explant weight increment was in a curvilinear relationship. We did observe a decreasing weight gain in HF210 after 28 days. Also based on Fig.2 and morphological observations, prolonged culture without subculturing could result in additional growth complications. Therefore, we would like to suggest a 21-day subculturing practice as optimal for Douglas-fir explants taking all the various factors and observations into account. We will provide a more precise conclusion in our revision.

# Peer review: Referee response for version 1

**DOI:** 10.5256/f1000research.6323.r7372

**Published:** 2015-02-06

**Authors:** Vibha Srivastava

**Affiliations:** 1 Department of Crop, Soil, and Environmental Sciences, University of Arkansas, Fayetteville, USA

This is a descriptive paper analyzing pH fluctuations in the tissue culture media for the micropropagation of Douglas fir, and assessing the effect of MES, a commonly used compound in tissue culture media, for stabilizing media pH. Parameters such as the effect of incubation time (days) and dark/light in the presence or absence of MES were studied, in addition to  the morphology of explant (buds) in MES + or - media. As expected, MES was found to be useful in stabilizing pH in different conditions and therefore recommended for the Douglas fir micropropagation media. This paper could be useful to the horticulturists, especially those working on micropropagation; though, the findings of this paper, in my opinion, are common knowledge in the field of tissue culture.

I have one major criticism on the experimental design and data analysis, which compels me to approve this paper with reservations: only one concentration, 2 g/L, of MES was used throughout this study. No reasoning was presented towards selection of this particular concentration. MES is used in many tissue culture media at different concentrations. While the authors shy away from making any conclusions (regarding the role of MES), they suggest MES played a role in stabilizing pH. This conclusion cannot be substantiated without a dose-response curve and determination of optimum concentration.

A minor criticism that I have is related to description in materials and methods. The tissue culture media used through out this study is simply referred by the acronym, mDCR. But no description of this media given is given, only reference provided. Whether it is MS/B5/N6 based media is not clear from this paper, and which other compounds are present in this media is not described.

I have read this submission. I believe that I have an appropriate level of expertise to confirm that it is of an acceptable scientific standard, however I have significant reservations, as outlined above.

# Response

**Published:** 2015-02-06

**Authors:** Chien-Chih Chen

**Affiliations:** Clemson University, USA

We would like to thank you for your comments on enhancing this manuscript. Once we received more reviews, we will include your suggestions and revise this manuscript.

# Response

**Published:** 2015-04-29

**Authors:** Chien-Chih Chen

**Affiliations:** Clemson University, USA

We appreciate your comments. Please find our explanations regarding your concerns with the original version (and how they have been addressed in the new version of the article) below.

1. We agree that MES is commonly used in tissue culture. It would have been better to establish a dose-response curve and determine the optimal concentration for our experimental system. But that would have involved testing the MES range with all of the starting pH levels and storage conditions and multiple explant genotypes.  However, we were limited in the number of explant samples available from the Christmas tree seed orchard, and over the course of the research we did not have enough material to investigate all of the possible multi-factorial combinations. Instead, since we were mostly interested in learning how medium pH changes under storage and culture conditions, and how that effects the explant growth responses, we started with one MES level previously reported with conifer explant culture. As expected, that level of MES did maintain a better medium pH. This study was at the beginning of a long series of experiments to establish a protocol for an effective micropropagation method for a recalcitrant woody species, Douglas-fir, for large scale application.  Because the one concentration of MES worked well, we then proceeded to a different set of experiments to test other factors involved in propagation and scale up.  Based on our findings in this study, we could have even chosen to omit the addition of MES altogether, by subculturing explants before the medium pH falls too low. In that case, we could reduce cost and perhaps establish a more economic micropropagation protocol for the industry.
2. The DCR medium itself is a modified version of the MS basal salts recipe developed for Douglas-fir by Gupta and Durzan (1985 and 1987). We further modified the DCR medium and classified it as mDCR, which is still based on MS. A comparison chart of the components between DCR vs. mDCR will be added in our revision.
